# Co-existence of two plasmids harboring transferable resistance-nodulation-division pump gene cluster, *tmexCD1-toprJ1*, and colistin resistance gene *mcr-8* in *Klebsiella pneumoniae*

**DOI:** 10.1186/s12941-024-00727-x

**Published:** 2024-07-26

**Authors:** Xiaofen Mo, Hui Zhang, Junfeng Fan, Linna Xu, Hao Fu, Junpeng Yue, Kaixuan Dong, Qixia Luo, Fen Wan

**Affiliations:** 1 School of Laboratory Medicine and Bioengineering, Hangzhou Medical College, Hangzhou, China; 2 State Key Laboratory for Diagnosis and Treatment of Infectious Diseases, Collaborative Innovation Center for Diagnosis and Treatment of Infectious Diseases, The First Affiliated Hospital of Medical School, College of Medicine, Zhejiang University, Hangzhou, China; 3 Key Laboratory of Biomarkers and In Vitro Diagnosis Translation of Zhejiang Province, Hangzhou, China; 4 The First Affiliated Hospital of Medical School, College of Medicine, Zhejiang University, Hangzhou, China

**Keywords:** *K. pneumoniae*, *mcr-8*, *tmexCD1-toprJ1*, Dissemination

## Abstract

**Background:**

The emergence of plasmid-mediated mobile colistin resistance (*mcr*) gene poses a great challenge to the clinical application of polymyxins. To date, *mcr-1* to *mcr-10* have been found in animals, humans, and the environment. Among them, *mcr-8* was first identified in *Klebsiella pneumoniae* (*K. pneumoniae*) of swine origin, and then *mcr-8.1* to *mcr-8.5* were successively identified. Notably, *K. pneumoniae* is the major host of the *mcr-8* gene in both animals and humans. This study aims to explore the characteristics of *K. pneumoniae* strains carrying the *mcr-8* gene and *tmexCD1-toprJ1* gene cluster and investigate the correlation between these two antibiotic resistance genes.

**Methods:**

The isolates from the poultry farms and the surrounding villages were identified by mass spectrometer, and the strains positive for *mcr-1* to *mcr-10* were screened by polymerase chain reaction (PCR). The size of the plasmid and the antimicrobial resistance genes carried were confirmed by S1-nuclease pulsed-field gel electrophoresis (S1-PFGE) and Southern hybridization, and the transferability of the plasmid was verified by conjugation experiments. Antimicrobial susceptibility testing (AST) and whole genome sequencing (WGS) were used to characterize the strains.

**Results:**

Two *K. pneumoniae* isolates (KP26 and KP29) displaying polymyxin resistance were identified as *mcr-8* gene carriers. Besides that, tigecycline-resistant gene cluster *tmexCD1-toprJ1* was also found on the other plasmid which conferred strain resistance to tigecycline. Through epidemiological analysis, we found that the *mcr-8* gene has dispersed globally, circulating in the human, animals, and the environment. Furthermore, our analysis suggests that the coexistence of *mcr-8* and *tmexCD1-toprJ1* on a single plasmid might evolved through plasmid recombination.

**Conclusions:**

Although the *mcr-8* and *tmexCD1-toprJ1* gene clusters in the two strains of *K. pneumoniae* in this study were on two different plasmids, they still pose a potential threat to public health, requiring close monitoring and further study.

**Supplementary Information:**

The online version contains supplementary material available at 10.1186/s12941-024-00727-x.

## Background

Multi-drug resistant (MDR) pathogens have become a vital global issue that caused by the overuse of antimicrobials in recent years [1]. Among MDR pathogens, *Klebsiella pneumoniae* is considered as the notorious recipient of multiple antimicrobial resistance (AMR) genes as hundreds of mobile AMR genes have been found in this bacterium [2]. Accumulation of AMR genes in *K. pneumoniae* is primarily the result of horizontal gene transfer (HGT) driven by plasmids and mobile genetic elements. The transmission of AMR genes in bacteria has posed a great threat to clinical and public health problems [3].

Polymyxins are a family of cyclic lipopeptides among which polymyxin B and polymyxin E (colistin) are the most used and studied variants. It acts as the last-resort antibiotics effective against infections caused by MDR Gram-negative bacteria in the clinical treatment. By electrostatic interacting with the negatively charged phosphate groups on lipid A of lipopolysaccharide (LPS) in the bacterial outer membrane, polymyxins inserted into the fatty acyl layer of the outer membrane and caused membrane disorganization and eventual cell death [4–7]. Since Liu et al. [8] reported for the first time the emergence of the plasmid-mediated polymyxin resistance gene *mcr-1* in *Enterobacteriaceae*, 10 *mcr* gene variants (*mcr-1* to *mcr-10*) were found in isolates sourcing from animals, environment, and humans over 20 countries [9–11]. The *mcr* gene mediates resistance by encoding a phosphoethanolamine (pEtN) transferase, which adds pEtN to the lipid A portion of LPS, thereby reducing the number of negatively charged phosphate groups on its surface. This modification results in a decreased affinity for polymyxin to bind to the target site, allowing for resistance to occur [10, 12]. A search of the GenBank database revealed that the *mcr-8* gene was mainly present in *K. pneumoniae* of human and animal origin [13]. Following the identification of a plasmid carrying *mcr-8.2* in *K. pneumoniae*, it has been speculated that animal-derived *Klebsiella* may be a reservoir for the *mcr-8* gene [14]. Gene *mcr-8* was identified on the transferable IncII plasmid of *K. pneumoniae*, and the acquisition of a single *mcr-8* gene significantly increased the colistin resistance of *Escherichia coli* (*E. coli*) and *K. pneumoniae* [13].

Tigecycline is a tetracycline class of antibacterial drugs that can be used to treat infections caused by polymicrobial MDR including both Gram-positive and Gram-negative bacteria [15]. The emergence of tigecycline resistance has been reported worldwide though few studies are available for deciphering the molecular mechanism of resistance to tigecycline. Recently, a novel plasmid-harbored resistance-nodulation-division (RND) efflux pump gene cluster, *tmexCD1-toprJ1,* was reported to mediate tigecycline resistance in Gram-negative bacteria [16, 17]. The global dissemination of plasmid-borne drug resistance genes threatens to compromise the efficacy of polymyxins and tigecycline, increasing the difficulty of treatment of associated bacterial infections.

This study aimed to investigate the mechanism of plasmid-mediated colistin and tigecycline resistance by analyzing the prevalence and genetic characteristics of the *mcr* gene and *tmexCD1-toprJ1* gene, underlining the co-transmission risk of these resistance genes.

## Methods

### Isolation and identification of strain and *mcr* genes

Samples of chicken manure, farm workers and healthy people were collected from chicken farms and their neighboring villages in Hangzhou. Fecal samples (≈1.0 g) were placed in 5 ml sterile Luria–Bertani broth and cultured overnight in an incubator at 37 ℃. The next day, the bacterial solution was coated on MacConkey agar for overnight culture. The MacConkey agar was repeatedly underlined to obtain a single colony. The species of bacteria were identified by matrix-assisted laser desorption ionization–time of flight mass spectrometry (MALDI-TOF MS), and the *mcr* genes of all isolates were subsequently sequenced by PCR and Sanger sequencing using specific primers [18]. Primer pairs used in this study were listed in Additional file 1: Table S1.

### Antimicrobial susceptibility testing

In accordance with Clinical and Laboratory Standards Institute (CLSI) guidelines, agar or broth microdilution is used to assess antimicrobial susceptibility. Microbroth dilution method was used to determine the MIC values of polymyxins (colistin and polymyxin B) and tigecycline, and agar dilution method was used to determine the MIC values of other antibiotics. Susceptibility results for all strains to tigecycline were interpreted according to the breakpoints of the European Committee for Antimicrobial Susceptibility Testing (EUCAST) (https://eucast.org/clinical_breakpoints/), and susceptibility results for other antibiotics were interpreted according to the CLSI guidelines. The AST was carried out using *E. coli* ATCC 25922 as the quality control standard.

### Conjugation experiments

To verify the transfer ability of plasmids carrying the *mcr-8* gene or *tmexCD1-toprJ1* gene cluster, we used strains carrying *mcr-8* or *tmexCD1-toprJ1* as donors, and sodium azide-resistant *E. coli* J53 and rifampicin-resistant EC600 as the recipient strains, respectively. Mueller–Hinton agar containing 200 mg/L sodium azide and 2 mg/L polymyxin B, 200 mg/L rifampicin and 4 mg/L tigecycline were used as the screening medium for transconjugants. We then selected transconjugants on Mueller–Hinton agar with sodium azide and polymyxin B, and detected the presence of the *mcr-8* gene in the transconjugants by PCR and DNA sequencing.

### S1-PFGE and southern hybridization

To identify the number and size of plasmids in *K. pneumoniae* carrying the *mcr-8* gene, we performed the S1-PFGE and Southern hybridization experiments. *Salmonella* H9812 was used as control strain and size marker [19]. After electrophoresis, it was hybridized with a digoxigenin-labeled *mcr-8* probe to determine the location of the *mcr-8* gene, and finally detected using the nitroblue tetrazolium–5-bromo-4-chloro-3-indolylphosphate (NBT-BCIP) color detection kit (Roche, catalog no. 11745832910).

### WGS and bioinformatic analysis

The genomic DNA of *mcr-8* positive isolates was extracted by Gentra Puregene yeast /Bact kit (Qiagen, CA, USA). WGS was then performed on the Nanopore PromethION platform (Nanopore, Oxford, UK) in accordance with the 10-kbp library protocol. Paired-end libraries detection was performed using the Illumina Novaseq 6000 system (Illumina, San Diego, CA, USA). Hybrid assembly of Illumina short reads and PromethION long reads using Unicycler v0.4.8. WGS data was annotated using rast (https://rast.nmpdr.org/). The assembled contigs were analyzed using the Center for Genomic Epidemiology website(https://www.genomicepidemiology.org/) to identify acquired antimicrobial resistance (AMR) genes, plasmid incompatibility types, and perform Multi Locus Sequence Typing (MLST). The analysis of virulence genes was performed on Virulence Factor Database (http://www.mgc.ac.cn/cgi-bin/VFs/v5/main.cgi).

The strains of *K. pneumoniae* carrying *mcr-8* were screened from NCBI nucleotide database by BLAST (https://blast.ncbi.nlm.nih.gov/Blast.cgi, accessed 29 March 2023), the *K. pneumoniae* harboring *mcr-8* in GenBank are shown in Additional file 2: Table S2. The phylogenetic tree was generated using Prokka and Roary. The transposon and insertion sequence were detected using the ISFinder database (https://isfinder.biotoul.fr/about.php). Finally, Easyfig.2.0 was used for linear comparison of multiple plasmids carrying *mcr-8* and *tmexCD1-toprJ1.*

## Results

### Characteristics of antimicrobial susceptibility of the *mcr*-positive *K. pneumoniae* strain

We collected fecal samples from chicken manure, chicken farm workers, and healthy workers in chicken farms and residents nearby villages around Hangzhou neighborhood. A total of 112 strains of *Enterobacteriaceae* were identified, among which 60 strains were isolated from chicken, 5 strains were from chicken farm workers, and 47 strains were from healthy residents. Colony PCR was conducted to screen *mcr* genes using primers as described in materials and methods. Two *K. pneumoniae* KP26 and KP29 from chicken manure were found to harbor *mcr-8* gene. Antimicrobial susceptibility analysis of KP26 and KP29 were performed and interpreted according to CLSI guidelines (Table 1). The result showed that KP26 and KP29 exhibited low-level resistance to polymyxin B (KP26, MIC = 4 mg/L; KP29, MIC = 4 mg/L) and colistin (KP26, MIC = 8 mg/L; KP29, MIC = 4 mg/L). Besides that, KP26 and KP29 also displayed resistance to tigecycline (KP26, MIC = 8 mg/L; KP29, MIC = 8 mg/L). MIC data revealed that KP26 and KP29 exhibited resistance to cefazolin (> 128 mg/L), levofloxacin (> 32 mg/L), aztreonam (64 mg/L), ceftriaxone (> 64 mg/L), cefepime (> 64 mg/L), amikacin (> 128 mg/L), ceftazidime (64 mg/L), cefepime-sulbactam (64/32 mg/L), and ciprofloxacin (> 32 mg/L), but is susceptible to carbapenem antibiotics, including ertapenem (0.25 mg/L), imipenem (0.125 mg/L), and meropenem (0.03 mg/L) (Table 1).

**Table 1:** Antimicrobial susceptibility testing of *mcr-8*-harboring isolates and their transconjugants

Antimicrobials	MIC (mg/L) of drug^a^				
	KP26	KP26/J53	KP29	KP29/J53	J53
CZO	> 128	4	> 128	4	4
MOX	2	0.25	2	0.25	0.25
LVX	> 32	0.06	> 32	0.06	0.06
ATM	64	0.125	64	0.125	0.125
CRO	> 64	0.06	> 64	0.06	0.06
FEP	16	≤ 0.015	16	≤ 0.015	≤ 0.015
AMK	> 128	2	> 128	2	2
CZA	0.5/4	0.25/4	0.5/4	0.25/4	0.25/4
CAZ	64	0.25	64	0.25	0.25
CSL	64/32	0.25/0.125	64/32	0.125/0.06	0.125/0.06
ETP	0.25	0.015	0.25	0.015	0.015
CIP	> 32	≤ 0.004	> 32	≤ 0.004	≤ 0.004
MEM	0.03	0.03	0.03	0.03	0.03
IMP	0.125	0.25	0.125	0.25	0.25
TGC	8	0.06	8	≤ 0.03	0.125
PMB	4	4	4	2	1
CST	8	4	4	4	1

^a^*CZO* cefazolin, *MOX*, latamoxef, *LVX* levofloxacin, *ATM* aztreonam, *CRO* ceftriaxone, *FEP* cefepime, *AMK* amikacin, *CZA* ceftazidime-avibactam, *CAZ* ceftazidime, *CSL* cefoperazone-sulbactam, *ETP* ertapenem, *CIP* ciprofloxacin, *MEM* meropenem, *IPM* imipenem, *TGC* tigecycline, *PMB* polymyxin B, *CST* colistin

**Fig. 1:**
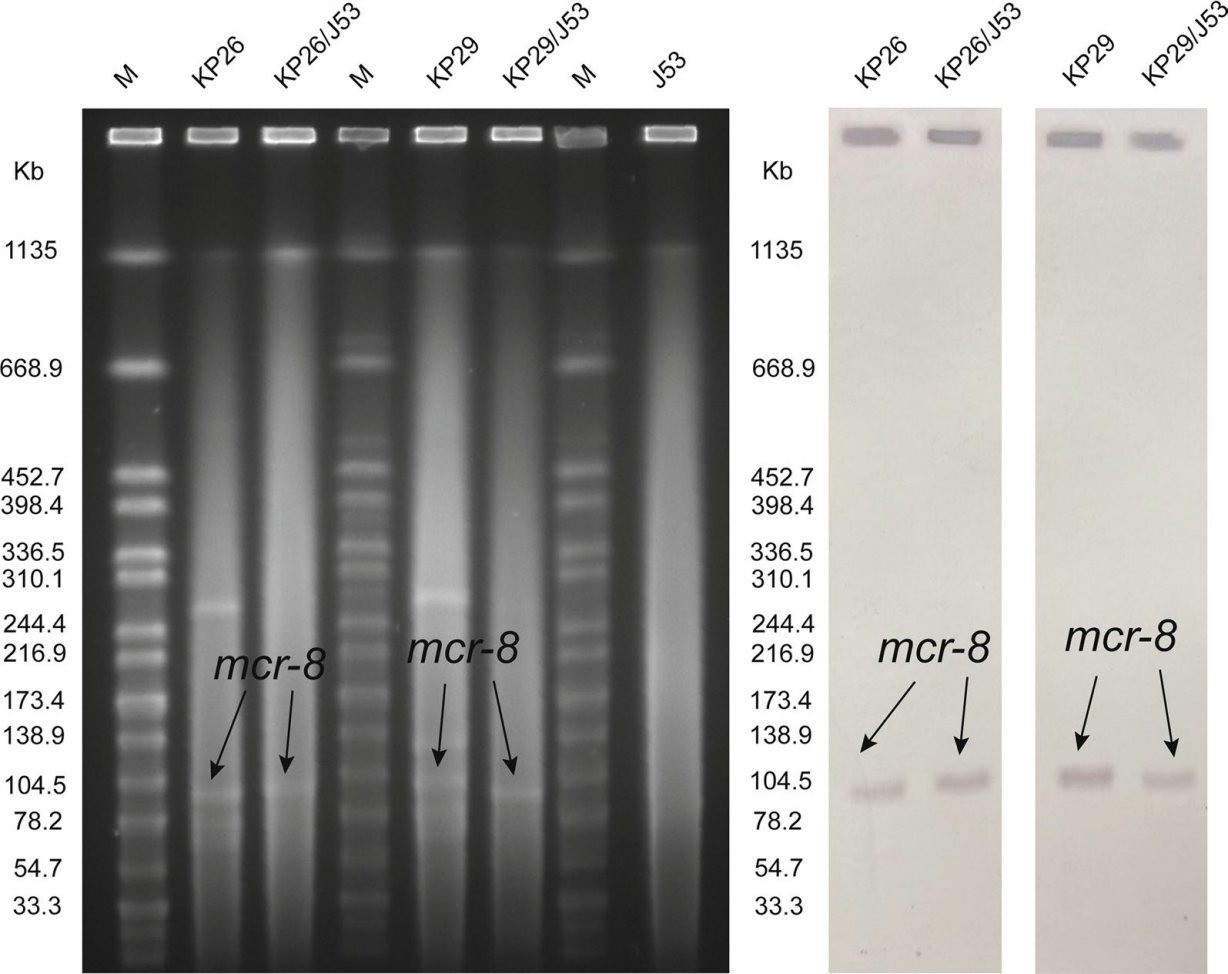
S1-PFGE profile (left) and Southern hybridization (right) analysis using *mcr-8*-specific probes to detect *mcr-8*-harboring isolates. *Salmonella* strain H9812 was used as a control strain and size marker. The names of the isolates are shown in the first line. The arrows indicated the locations of *mcr-8* harboring plasmids according to the Southern hybridization experiment

Conjugation experiments showed that the colistin resistance gene *mcr-8* of KP26 and KP29 could be successfully transferred to the recipient *E. coli* J53, making the conjugants acquire colistin resistance phenotype. Results of S1-PFGE and Southern hybridization showed that KP26 carried 4 plasmids and KP29 carried 5 plasmids, with *mcr-8* gene located at plasmid 101.2 kb (Fig. 1).

### Genomic analysis and molecular type of *K. pneumonia* KP26 and KP29

The complete genomes of isolates KP26 and KP29 were sequenced by WGS. KP26 consists of a 5,252,651 bp chromosome and four plasmids (pKP26-tmexCD1, pKP26-mcr8, pKP26-3, pKP26-4) (Table 2). KP29 consists of a 5,253,463 bp chromosome and five plasmids (pKP29-tmexCD1, pKP29-2, pKP29*-*mcr8, pKP29-4, pKP29-5) (Table 2). MLST analysis showed that the KP26 and KP29 strains belong to ST11.

**Table 2:** Features of the isolates

Isolate	Source	DNA types	AMR gene	MLST/plasmid replicon	DNA size(bp)
KP26	Chicken	Chromosome	*bla*_*SHV-182*_*, fosA, oqxB, oqxA*	ST11	5,252,651
		pKP26-tmexCD1	*aph(6)-Id, aph(3')-Ia, aph(3'')-Ib, aadA1, aadA2b, armA, bla*_*DHA-1*_*, msr(E), mph(E), cmlA1, qnrB4, sul1, sul3, tmexD1, toprJ1, tmexC1*	IncFIB(Mar)/IncHI1B	271,379
		pKP26-mcr8	*mcr-8*	IncFIA/IncFII	101,185
		pKP26-3	*aac(3)-IId, aadA16, aph(6)-Id, aph(3')-Ia, aph(3'')-Ib, aac(6')-Ib-cr, mph(A), ARR-3, sul1, sul2, tet(A), dfrA27*	IncFIA,IncQ1,IncR	83,074
		pKP26-4	*bla*_*CTX-M-15*_*, bla*_*TEM-1B*_*, qnrS1*	–^a^	72,637
KP29	Chicken	Chromosome	*bla*_*SHV-182*_*, fosA, oqxB, oqxA,*	ST11	5,253,463
		pKP29-tmexCD1	*aph(6)-Id, aph(3')-Ia, aph(3'')-Ib, aadA1, aadA2b, armA,bla*_*DHA-1*_*, msr(E), mph(E), cmlA1, qnrB4, sul1, sul3, tmexD1, toprJ1, tmexC1*	IncFIB(Mar)/IncHI1B	271,379
		pKP29-2	*floR, qnrB52, sul1, tet(A)*	IncFIB	131,420
		pKP29-mcr8	*mcr-8*	IncFIA/IncFII	101,185
		pKP29-4	*aac(3)-IId, aadA16, aph(6)-Id, aph(3')-Ia, aph(3'')-Ib, aac(6')-Ib-cr, mph(A), ARR-3, sul1, sul2, tet(A), dfrA27*	IncFIA,IncQ1,IncR	83,068
		pKP29-5	*bla*_*CTX-M-15*_*, bla*_*TEM-1B*_*, qnrS1*	–^a^	72,637

^a^No resistance genes or plasmid replicon was found

Antibiotic resistance determinants *bla*_*SHV-182*_, *fosA*, *oqxA* and *oqxB* were both detected on chromosomes of KP26 and KP29. Most of the resistance genes are located on plasmids, especially on the large plasmids pKP26-tmexCD1 and pKP29-tmexCD1, which are 271,379 bp. They include *aph(6)-Id*, *aph(3')-Ia*, *aph(3'')-Ib*, *aadA1*, *aadA2b*, *armA*, *bla*_*DHA-1*_, *msr(E)*, *mph(E)*, *cmlA1*, *qnrB4*, *sul1*, *sul3*, *tmexD1*, *toprJ1*, and *tmexC1*. Only one resistance gene, *mcr-8*, has been identified in pKP26-mcr8 and pKP29-mcr8. Other resistance genes were identified on two other plasmids of KP26 and three other plasmids of KP29.

### Characterization of *mcr-8-*harboring plasmids

**Fig. 2:**
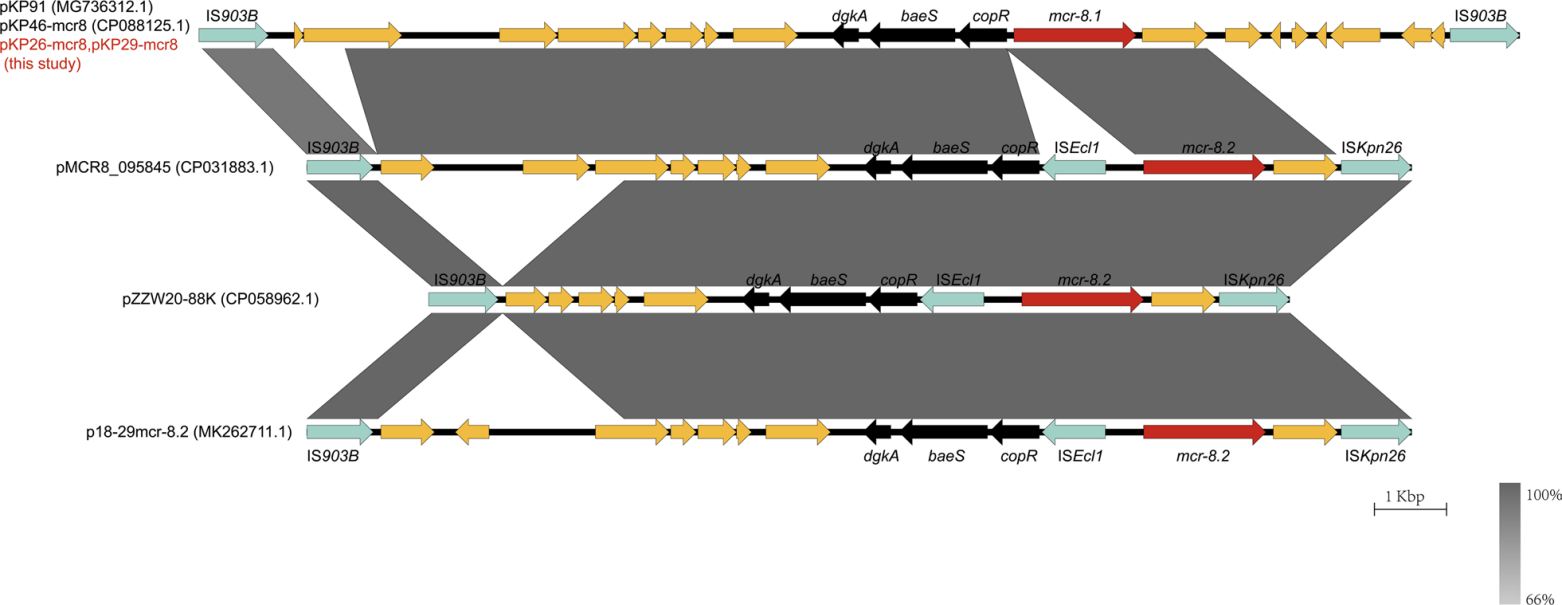
Genetic features of the *mcr-8*-carrying plasmid in *K. pneumoniae* strain KP26 and KP29. The linear genetic environment surrounding *mcr-8* is depicted. Arrows indicate the direction of transcription for each gene, and different colors represent different genes. The *mcr-8* gene is shown in red, genes associated with mobile elements are shown in green, the three genes in the figure are shown in black, and hp and other functional genes are shown in yellow. Regions with high homology are represented by gray shading

Plasmid pKP26-mcr8 was of type IncFIA/IncFII with a total length of 101,185 bp and contained only one antimicrobial resistance gene, *mcr-8.1*. It was 100% identical to plasmid pKP29-mcr8. Therefore, we only describe the characterization of pKP26-mcr8. The *mcr-8.1* gene on pKP26-mcr8 was associated with the genetic context of IS*903B*-orf-*dgkA*-*baeS*-*copR*-*mcr-8.1*-orf-IS*903B*. The pKP26-mcr8 was shown to be conjugative under laboratory conditions. We performed comparative genomic analysis based on DNA sequences of pKP26-mcr8 and five plasmids with different sources in NCBI (Fig. 2). The results showed that pKP26-mcr8, pKP46-mcr8 and pKP91 [13] isolated from animals had similar genetic background around the *mcr-8* gene IS*903B*-orf-*dgkA*-*baeS*-*copR*-*mcr-8.1*-orf-IS*903B*. In clinical plasmid pMCR8_095845, p18-29mcr-8.2 and environmental plasmid pZZW20-88 K, IS*Kpn26* took the position of IS*903B,* which was located in the downstream region of *mcr-8* gene.

### Evolutionary analysis of isolates carrying *mcr-8*

**Fig. 3:**
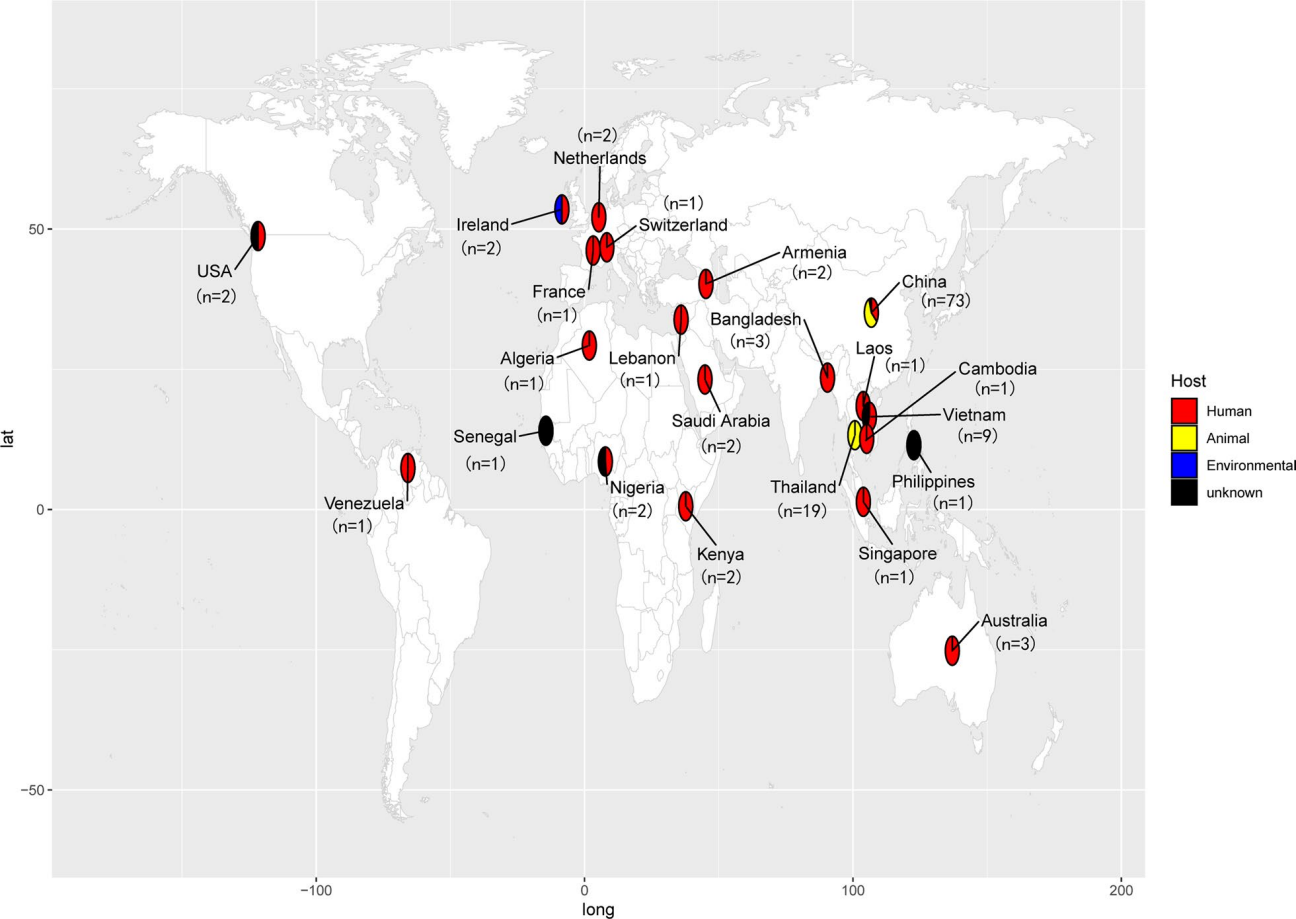
Among all 138 isolates containing *mcr-8* in the NCBI database, 131 of them had geographical distribution with well-defined locations. Different hosts of the isolates are marked with different colors, with red, yellow, blue and black indicating human, animal, environmental and unknown origin respectively. The number of isolates harboring *mcr-8* in each country is plotted under the country name on a world map. And the world map was created using the corresponding map data of the R package ggplot2 v3.3.5

**Fig. 4:**
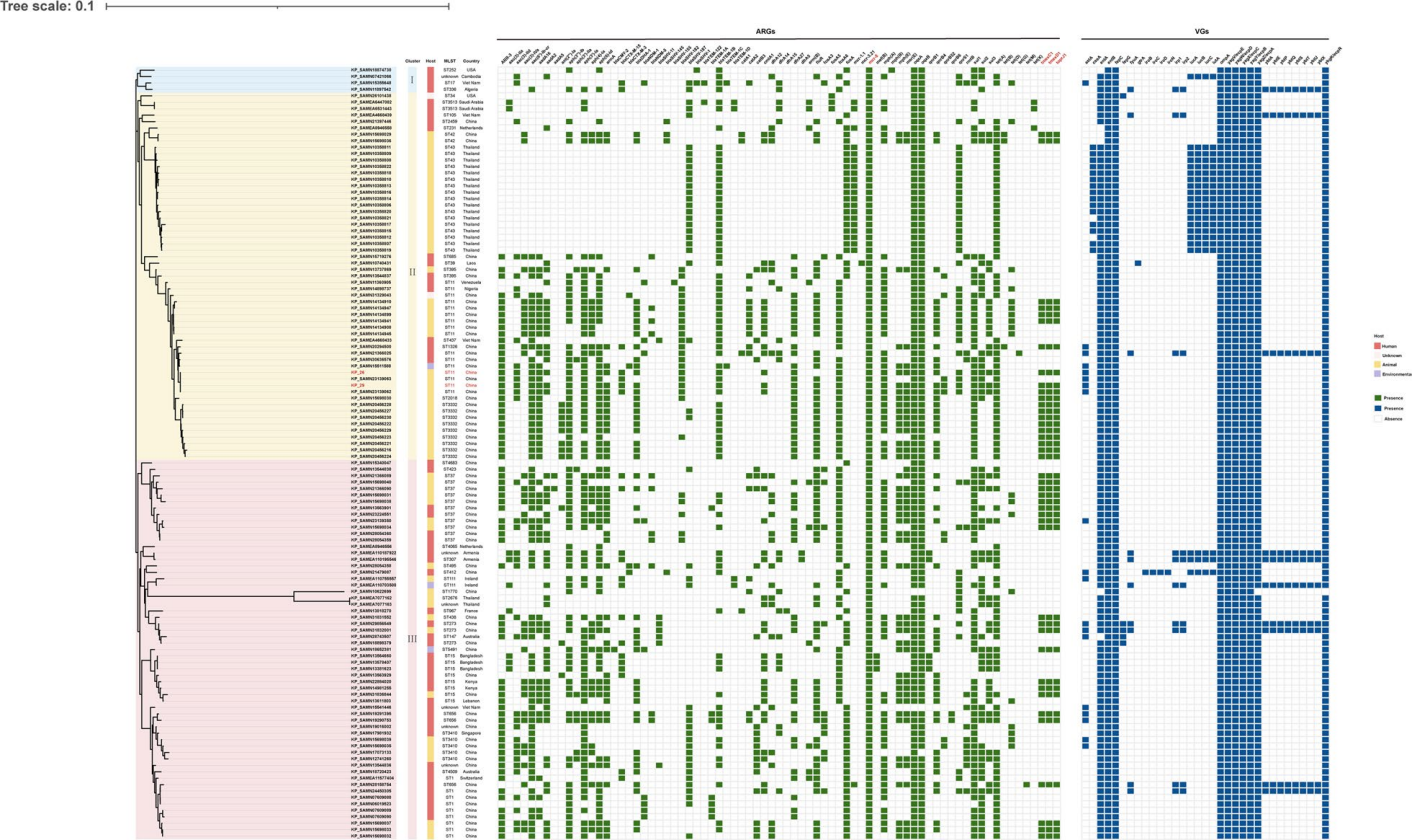
Phylogenetic tree generated from the core genome sequences of the *mcr-8*-harboring isolates identified in this study and other *mcr-8*-harboring *K. pneumoniae* isolates in the NCBI database. Each isolate has a sample identifier (ID), location, MLST, and host. The figure shows important antimicrobial resistance genes (ARGs) and major virulence-associated genes (VGs)

We retrieved genome sequences of 138 *mcr-8* positive *K. pneumoniae* from the NCBI database. Among these isolates, 122 strains had definite source. There are 63 strains isolated from animals, 56 strains from human, and 3 from environment (Fig. 3). Twenty strains did not have complete genome, so we constructed a phylogenetic tree consisting of 118 *K. pneumoniae* isolates, *K. pneumoniae* KP26 and KP 29 based on single nucleotide polymorphisms (SNPs) of core genomes. The 120 *K. pneumoniae* isolates were clustered in 3 clonal groups (Fig. 4). These isolates were identified in North America, Asia and Africa. Most of them were detected in China, followed by Thailand and Vietnam, indicating that Asia is the main prevalent continent for *mcr-8* positive *K. pneumoniae*. The most common MLST type among the 114 strains was ST43 (17/114), followed by ST11 (16/114), which is also the dominant epidemic strain in China [20]. *K. pneumoniae* KP26 and KP29 were belong to cluster II and closed to KP_SAMN23139063. It is worth noting that isolates in cluster III were from different countries around world isolated from humans, animals and environment. This implies that *mcr-8* positive *K. pneumoniae* are undergoing evolution during the process of dissemination.

In order to investigate the virulence genes in *mcr-8*-positive *K. pneumoniae,* the whole genome sequence of *mcr-8*-positive *K. pneumoniae* were compared with virulence factor database VFDB. The results showed that the virulence genes included iron-absorption virulence gene (*entA*, *entB*, *fepC*), invasive virulence genes (*ompA*), adhesive virulence gene (*yagV*/*ecpE*, *yagW*/*ecpD*, *yagX*/*ecpC*, *yagY*/*ecpB*, *yagZ*/*ecpA*, *ykgK*/*ecpR*), and enterotoxin gene (*astA*). Among the 120 strains of *K. pneumoniae*, only 1 strain was found to carry both *iroB* and *iucA*.

### Characterization of the *tmexCD1-toprJ1-*harboring plasmid

**Fig. 5:**
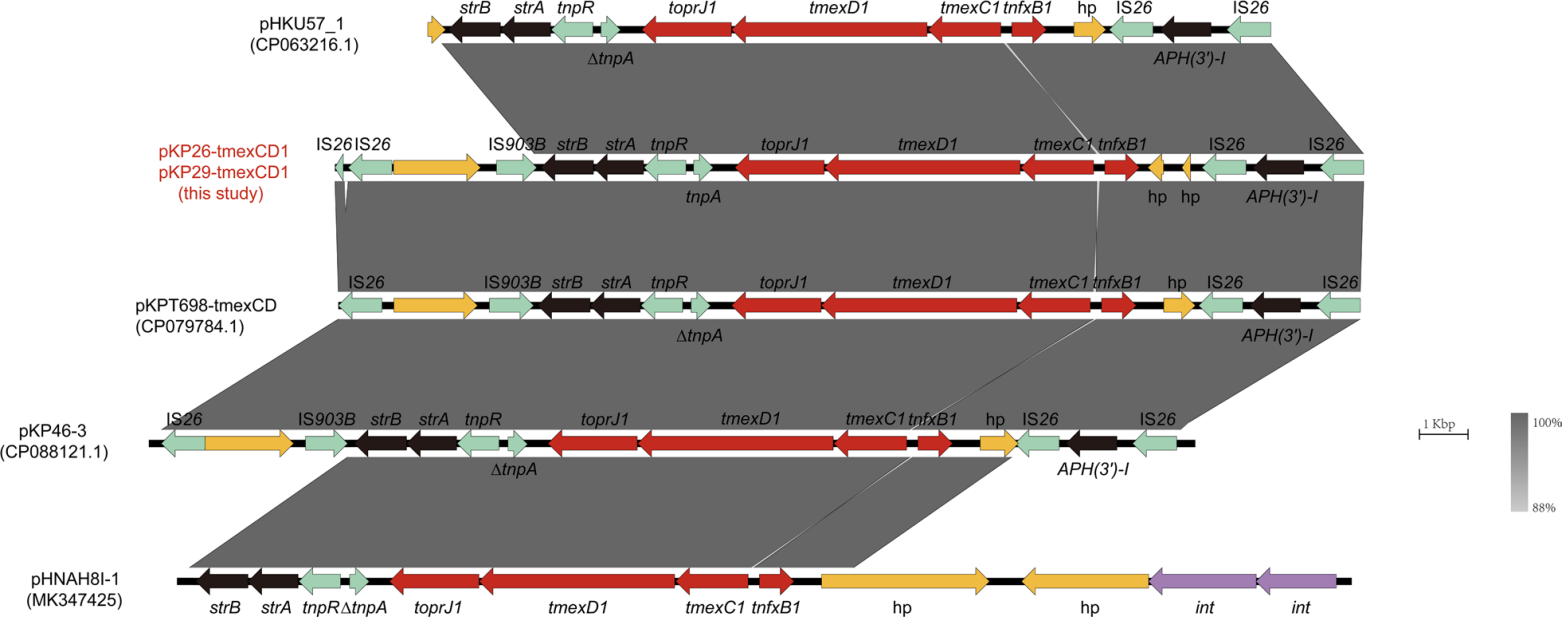
Genetic features of the *tmexCD1-toprJ1*-carrying plasmid in *K. pneumoniae* strain KP26 and KP29. The linear genetic environment surrounding *tmexCD1-toprJ1* is depicted. Arrows indicate the direction of transcription of each gene, and different genes are shown in different colors. The *tmexCD1-toprJ1* gene is shown in red, genes associated with mobile elements are shown in green, the three genes in the figure are shown in black, hp and other functional genes are shown in yellow, and *int* genes are shown in purple

The plasmid type of pKP26-tmexCD1 was IncFIB(Mar)/IncHI1B, with a length of 271,379 bp. Resistance genes include *aph(6)-Id*, *aph(3’)-Ia*, *aph(3’)-Ib*, *aadA1*, *aadA2b*, *armA*, *bla*_*DHA-1*_, *msr(E)*, *mph(E)*, *cmlA1*, *qnrB4*, *sul1*, and *sul3*. The sequence of pKP29-tmexCD1 was 100% identical to pKP26-tmexCD1. To investigate the core genetic environment of *tmexCD1-toprJ1*, we analyzed the *tmexCD1-toprJ1-*containing regions from four *tmexCD1-toprJ1-*bearing plasmids in *Klebsiella* spp. isolated from animals, humans and environment. The core genetic structures of *tmexCD1-toprJ1* in *Klebsiella* spp. were divided into two types (Fig. 5). The type I genetic context is IS*903B*-*strB*-*strA*-*tnpR*-*∆tnpA*-*toprJ1*-*tmexD1*-*tmexC1*-*tnfxB1*-*∆hp*-IS*26*. Four *tmexCD1-toprJ1-*bearing plasmids (pHKU57_1, pKP26-tmexCD1, pKP46-3, and pKPT698-tmexCD) were belong to type I, and slightly different is that the pHKU57_1 lacks an IS*903B* upstream. They possessed two insertion sequence (IS) elements IS*26* located on the flank of *tmexCD1-toprJ1,* which conferred the ability to acquire and mobilize *tmexCD1-toprJ1*, resulting the transmission in *Klebsiella*. The core genetic environment of type II was *strB*-*strA*-*tnpR*-*ΔtnpA*-*toprJ1*-*tmexD1*-*tmexC1*-*tnfxB1*-*hp* in plasmid pHNAH8I-1[16]. It lacked the flanked IS*26* element compared with type I, which indicates that the mobilization of *tmexCD1-toprJ1* might be driven by IS*26* from the plasmid pHNAH8I-1[21]. The IS elements play an important role in the spreading of *tmexCD1-toprJ1*.

## Discussion

In this study, we found that two strains of *K. pneumoniae* isolated from chickens were identified as belonging to multi-drug resistant ST11 type and carried both the *mcr-8* gene and the *tmexCD1-toprJ1* gene cluster on two different plasmids. The complete genome sequences of two strains were obtained by WGS and compared with the genome of other *K. pneumoniae* isolates carrying the *mcr-8* gene and *tmexCD1-toprJ1* retrieved from NCBI database. Our results showed that *mcr-8* gene is globally dispersed and has been circulating in the human, animals, and the environment, urging us to pay close attention to the convergence and co-transmission of *mcr-8* and the *tmexCD1-toprJ1* gene in *K. pneumoniae.*

In recent years, the discovery of *mcr* genes carried by plasmids has attracted a great deal of public attention. To date, a total of ten *mcr* genes (*mcr-1* to *mcr-10*) have been identified [10]. The *mcr-8* gene was initially detected on an IncFII-type plasmid carrying carbapenemase-encoding gene *bla*_*NDM*_ in swine-origin *K. pneumoniae.* Although *mcr-8* gene and its variants have been identified in humans, animals, and environmental sources of *K. pneumoniae*, *Raoultella ornithinolytica*, and *Klebsiella quasipneumoniae*, *K. pneumoniae* is still considered as the primary host for the *mcr-8* gene in both animals and human [13, 14, 22, 23]. In this study, we collected 112 strains of *Enterobacteriaceae* isolated from chicken manure and identified two *mcr-8*-positive *K. pneumoniae* strains. WGS revealed that these two strains also carried the tigecycline resistance gene cluster *tmexCD1-toprJ1*. The genetic context of *mcr-8* in pKP26-mcr8 and pKP29-mcr8 is flanked with IS*903B*. This arrangement is consistent with genetic context of the *mcr-8* gene in pKP91 [13]. But in the environmental isolate pZZW20-88 K, a single IS*903B* is located upstream of the *mcr-8* gene, while IS*Kpn26* is located downstream. Similarly, in the human isolate pMCR8_095845, IS*Kpn26* lies in the downstream region of *mcr-8* gene. Therefore, we speculated that the insertion sequence flanked with *mcr-8* gene is variable and both IS*903B* and IS*Kpn26* can mediate the transference of *mcr-8* gene.

We also analyzed the nucleotide and protein sequences of genes that are reported to be involved in polymyxin resistance, including *mgrB*, *phoP*/*phoQ*, *pmrA*/*pmrB*, *crrA*/*crrB*, *qseB*/*qseC*, *yciM* and *lpxM* in KP26 and KP29. In addition to *crrA/crrB* genes which were not exist, two type of point mutations were found in the *lpxM* of KP26 and KP29 (I7N and S229T). Whether *lpxM* mutations affects the polymyxins resistance in KP26 and KP29 needs further investigation.

The *tnfxB1*-*tmexCD1*-*toprJ1* gene cluster was found on the transposon Tn5393. There are two IS*26* located upstream and downstream of this gene cluster, indicating that the *tmexCD1-toprJ1* gene cluster was transferred via IS*26* transposition [21]. In addition, we found that pKP26-tmexCD1 contain a *umuC* gene serving as the integration site for the mobilization of *tmexCD1-toprJ1* gene cluster mediated by *int* gene. However, attempts to transfer the plasmid carrying this gene cluster into EC600 through conjugation experiments or electroporation, were unsuccessful. We inferred that the *tmexCD1-toprJ1-*carrying plasmid might have a preference for the host. This might explain the widespread presence of this gene cluster in *K. pneumoniae*, which is rare in other *Enterobacteriaceae* species [24]. Apart from chicken in this study, the *tmexCD1-toprJ1* was also detected in ST6265 *K. pneumoniae* strains from patient sources [25]. This result suggests that strains carrying *tmexCD1-toprJ1* are capable of transmitting between human and animals. We also investigated the existence of iron-absorption virulence gene, invasive virulence gene and adhesive virulence gene in KP26 and KP29 through WGS analysis. However, hypervirulence genes were not found in these two strains. This indicates that KP26 and KP29 are not hypervirulent strains.

Although our study found that the *mcr-8* and *tmexCD1-toprJ1* gene were located on the two different plasmids, it has been reported that the *mcr-8* gene and the *tmexCD1-toprJ1* gene cluster can coexist on the same plasmid and undergo co-transfer during transmission. Plasmid recombination have occurred during the conjugation between the *mcr-8-*carrying plasmid and *tmexCD1-toprJ1*-carrying plasmid mediated by IS*26* [26]. Using colistin could accelerate the process of plasmid fusion and formation of tigecycline-resistant strains. Through phylogenetic analysis of KP26, KP29, and *mcr-8*-carrying *K. pneumoniae* strains from the NCBI database, it was observed that the co-occurrence of the *tmexCD1-toprJ1* gene cluster was found in 35% (42/120) of the strains. Therefore, it is highly possible that IS*26* can mediate the plasmid fusion between pKP26-tmexCD1 and pKP26-mcr8 to carry more antibiotic resistance genes. The emergence of isolates resistant to both colistin and tigecycline is inevitable due to the use of these drugs as last-resort antibiotic treatments. This, in turn, facilitates the spreading of *mcr-8* and *tmexCD1-toprJ1* within the same strain.

## Conclusions

In conclusion, we reported the presence of the *mcr-8* and *tmexCD1-toprJ1* gene cluster in two animal-derived *K. pneumoniae* strains in the chicken farm in China. These two resistance genes were located on two different plasmids. By screening *mcr-8*-carrying *K. pneumoniae* isolates from the NCBI database, we found *mcr-8* positive *K. pneumoniae* strains have distributed around the world, spreading in different hosts. The acquisition of colistin and tigecycline resistance genes by *K. pneumoniae* might pose a serious threat to the public health. Therefore, we emphasize the importance of a “One Health” approach, particularly enhancing surveillance for the coexistence of *mcr-8* and *tmexCD1-toprJ1* in the same *K. pneumoniae* strain.

### Supplementary Information

Supplementary Material 1. The primer sequence of *mcr-1* to *mcr-10*.docx

Supplementary Material 2.* Klebsiella pneumoniae* harboring *mcr-8* in GenBank.xls

Supplementary Material 3. The figures mentioned in the main text.

## Data Availability

The complete sequence of KP29 and KP26 has been submitted to GeneBank under accession no. CP140760 to CP140770.

## Abbreviations

*K. pneumoniae*: *Klebsiella pneumoniae*
*E. coli*: *Escherichia coli*
PCR: Polymerase chain reaction
S1-PFGE: S1-nuclease pulsed-field gel electrophoresis
AST: Antimicrobial susceptibility testing
WGS: Whole genome sequencing
MALDI-TOF–MS: Matrix-assisted laser desorption ionization time-of- flight mass spectrometry
CLSI: Clinical and Laboratory Standards Institute
EUCAST: European Committee on Antimicrobial Susceptibility Testing
MIC: Minimum inhibitory concentration
MDR: Multi-drug resistant
AMR: Antimicrobial resistance
RND: Resistance-Nodulation-Division
LPS: Lipopolysaccharide
MLST: Multi Locus Sequence Typing
NBT-BCIP: Nitroblue tetrazolium–5-bromo-4-chloro-3-indolylphosphate
