# Supplementary material for: Co-existence of two plasmids harboring transferable resistance-nodulation-division pump gene cluster, tmexCD1-toprJ1, and colistin resistance gene mcr-8 in Klebsiella pneumoniae
Source: Ann Clin Microbiol Antimicrob. 2024 Jul 26;23:67. doi: 10.1186/s12941-024-00727-x (PMC11282740; doi:10.1186/s12941-024-00727-x)
Supplement: Supplementary file 1 — Supplementary Material 1. The primer sequence of mcr-1 to mcr-10.docx [file 12941_2024_727_MOESM1_ESM.zip › Additional file 1 Table S1/The primer sequence of mcr-1 to mcr-10.docx.docx]

**Supplementary Table S1**

**The primer sequence of *mcr-1* to *mcr-10*.**

| gene | primer | sequence | product (bp) | reference |
| --- | --- | --- | --- | --- |
| *mcr1* | mcr1_F | TCCAAAATGCCCTACAGACC | 205 | Xu L et al.,2022 |
|  | mcr1_R | GCCACCACAGGCAGTAAAAT |  |  |
| *mcr2* | mcr2_F | CCTTTTGTGCTGATGGGTTT | 279 | Xu L et al.,2022 |
|  | mcr2_R | ATTTTGGAGCATGGTGGTGT |  |  |
| *mcr3* | mcr3_F | CTTGCTGAACCAATCCCATT | 347 | Xu L et al.,2022 |
|  | mcr3_R | CCATCGTTCTCCTTCCACAAAA |  |  |
| *mcr4* | mcr4_F | GATCCGAAGCTGTGTTCTG | 426 | Xu L et al.,2022 |
|  | mcr4_R | GCCAGCATTGGTACGCTAGT |  |  |
| *mcr5* | mcr5_F | GGTTGGCCGAGAAGATAACA | 522 | Xu L et al.,2022 |
|  | mcr5_R | ATGTTGCCAGAAGGTCCAAC |  |  |
| *mcr6* | mcr6_F | AGCTATGTCAATCCCGTGAT | 252 | Borowiak et al.,2020 |
|  | mcr6_R | ATTGGCTAGGTTGTCAATC |  |  |
| *mcr7* | mcr7_F | GTCAGTTACGCCATGCTCAA | 791 | Xu L et al.,2022 |
|  | mcr7_R | TTCTTGTCGCAGAACTGTGG |  |  |
| *mcr8* | mcr8_F | AAACTGAACCCGGTACAACG | 943 | Xu L et al.,2022 |
|  | mcr8_R | GCCATAGCACCTCAACACCT |  |  |
| *mcr9* | mcr9_F | GCGGTTGTAAAGGCGTATGT | 635 | Xu L et al.,2022 |
|  | mcr9_R | CAAATCGCGGTCAGGATTAT |  |  |
| *mcr10* | mcr10_F | GGACCGACCTATTACCAGCG | 366 | Lei et al.,2020 |
|  | mcr10_R | GGCATTATGCTGCAGACACG |  |  |

**Reference**

Xu L, Fan J, Fu H, Yang Y, Luo Q, Wan F. The variants of polymyxin susceptibility in different species of genus *Aeromonas*. Front Microbiol. 2022 Oct 25;13:1030564. doi: 10.3389/fmicb.2022.1030564. PMID: 36386612; PMCID: PMC9642839.

Borowiak M, Baumann B, Fischer J, Thomas K, Deneke C, Hammerl JA, Szabo I, Malorny B. Development of a Novel *mcr-6* to *mcr-9* Multiplex PCR and Assessment of *mcr-1* to *mcr-9* Occurrence in Colistin-Resistant *Salmonella enterica* Isolates From Environment, Feed, Animals and Food (2011-2018) in Germany. Front Microbiol. 2020 Feb 4;11:80. doi: 10.3389/fmicb.2020.00080. PMID: 32117115; PMCID: PMC7011100.

Lei CW, Zhang Y, Wang YT, Wang HN. Detection of Mobile Colistin Resistance Gene *mcr-10.1* in a Conjugative Plasmid from *Enterobacter roggenkampii* of Chicken Origin in China. Antimicrob Agents Chemother. 2020 Sep 21;64(10):e01191-20. doi: 10.1128/AAC.01191-20. PMID: 32718964; PMCID: PMC7508621.
