# Supplementary figures and images for: Co-existence of two plasmids harboring transferable resistance-nodulation-division pump gene cluster, tmexCD1-toprJ1, and colistin resistance gene mcr-8 in Klebsiella pneumoniae
Source: Ann Clin Microbiol Antimicrob. 2024 Jul 26;23:67. doi: 10.1186/s12941-024-00727-x (PMC11282740; doi:10.1186/s12941-024-00727-x)

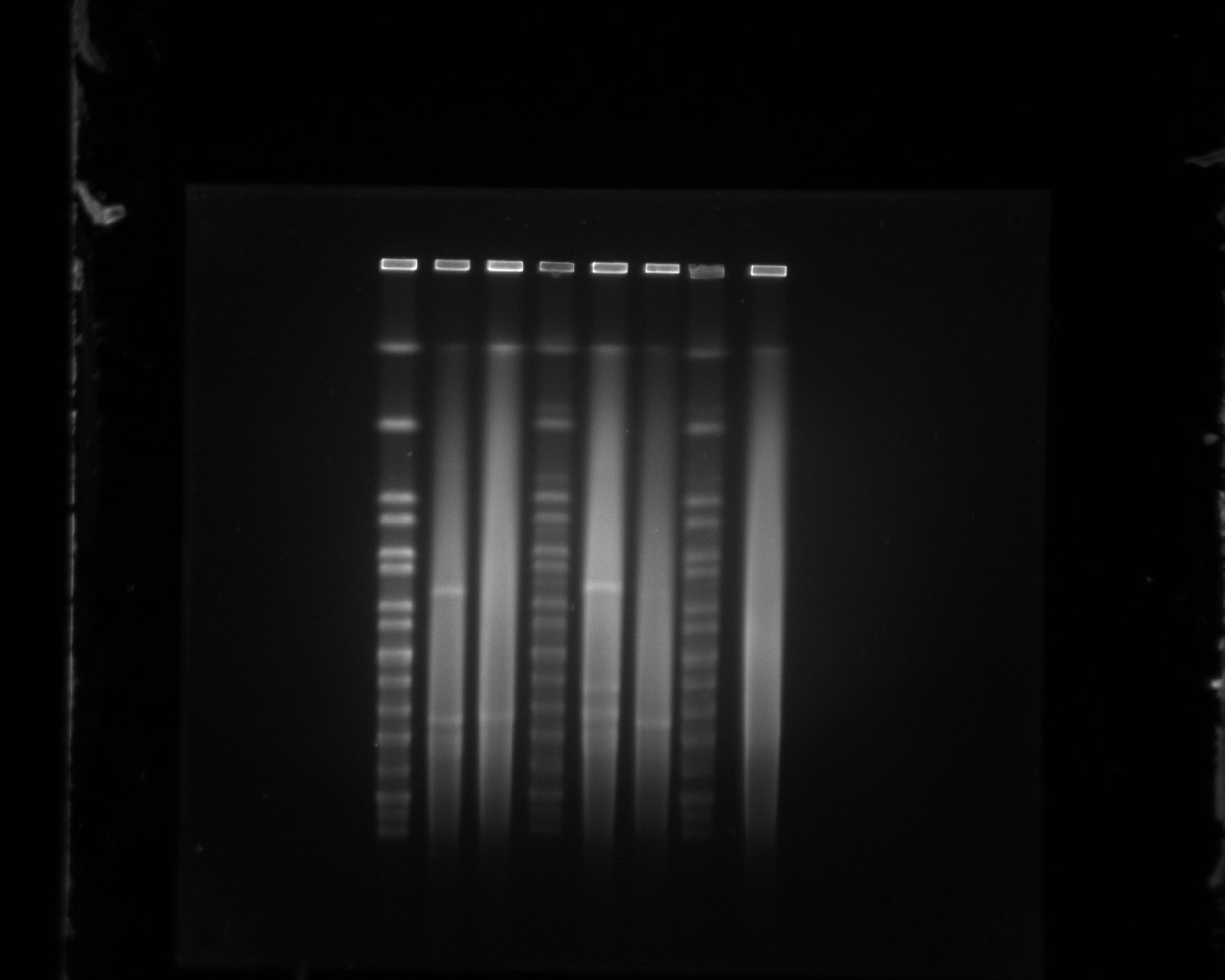

Supplement: Supplementary file 3 — Supplementary Material 3. The figures mentioned in the main text. [file 12941_2024_727_MOESM3_ESM.zip › Additional file 3 Figures/S1-PFGE.Tif]
